# Lightweight Dual-Attention Network for Concrete Crack Segmentation

**DOI:** 10.3390/s25144436

**Published:** 2025-07-16

**Authors:** Min Feng, Juncai Xu

**Affiliations:** 1 Anhui Provincial International Joint Research Center of Data Diagnosis and Smart Maintenance on Bridge Structures, Chuzhou 239099, China; minfeng@njmu.edu.cn; 2 Nanjing Rehabilitation Medical Center, Nanjing Medical University, Nanjing 210029, China; 3 College of Water Conserwancy and Hydropower Engineering, Hohai University, Nanjing 210098, China

**Keywords:** structural health monitoring, crack segmentation, dual-attention network, edge computing, real-time inference

## Abstract

Structural health monitoring in resource-constrained environments demands crack segmentation models that match the accuracy of heavyweight convolutional networks while conforming to the power, memory, and latency limits of watt-level edge devices. This study presents a lightweight dual-attention network, which is a four-stage U-Net compressed to one-quarter of the channel depth and augmented—exclusively at the deepest layer—with a compact dual-attention block that couples channel excitation with spatial self-attention. The added mechanism increases computation by only 19%, limits the weight budget to 7.4 MB, and remains fully compatible with post-training INT8 quantization. On a pixel-labelled concrete crack benchmark, the proposed network achieves an intersection over union of 0.827 and an F1 score of 0.905, thus outperforming CrackTree, Hybrid 2020, MobileNetV3, and ESPNetv2. While refined weight initialization and Dice-augmented loss provide slight improvements, ablation experiments show that the dual-attention module is the main factor influencing accuracy. With 110 frames per second on a 10 W Jetson Nano and 220 frames per second on a 5 W Coral TPU achieved without observable accuracy loss, hardware-in-the-loop tests validate real-time viability. Thus, the proposed network offers cutting-edge crack segmentation at the kiloflop scale, thus facilitating ongoing, on-device civil infrastructure inspection.

## 1. Introduction

Concrete surface cracking is an early, reliable indicator of reinforcement corrosion, moisture ingress, and the progressive loss of load-bearing capacity [1,2,3]. When fissures go undetected, deterioration accelerates, thus shortening the residual service life of bridges, tunnels, and pavements and inflating maintenance budgets [4,5]. Although current regulations mandate periodic visual surveys, these inspections demand scaffolding, lane closures, and expert judgement, so both temporal resolution and spatial coverage remain limited. Critical defects may therefore persist unnoticed for months [6,7]. Vision-based automatic crack assessment can close this monitoring gap by enabling continuous, non-contact surveillance with fixed cameras or aerial drones [8,9,10].

Classical computer vision pipelines include edge detection operators, intensity thresholding, morphological filtering, and the influential CrackTree framework, which integrates geodesic shadow removal with tensor voting [11]. These methods perform well on laboratory imagery but degrade sharply under field conditions characterised by non-uniform illumination, textured backgrounds, and sub-millimetre cracks [12,13]. Deep learning has improved robustness [14], yet most state-of-the-art networks rely on heavy backbones (e.g., ResNet-101 in DeepLabV3+) [15] or detection heads (e.g., the YOLO family) [16], whose memory footprints and power budgets exceed the constraints of watt-level edge devices [17,18]. The lightweight YOLOv5-DE integrates dense feature connections and dual attention to detect millimetre-level cracks with high accuracy at ~296 FPS, but its ultrafast predictions yield bounding boxes [19]. Lightweight variants based on MobileNetV3 or ESPNet [20] alleviate these costs only partially; they often misclassify rebar shadows and surface stains and fail to resolve crack width faithfully [21,22]. The naïve integration of attention modules, such as CBAM (sequential channel–spatial recalibration) [23] or DANet (dual self-attention) [24], sharpens feature activations but inflates parameter counts and inference latency [25,26].

To reconcile pixel-level precision with strict resource limits, this study introduces L-DANet, which is a lightweight dual-attention network for crack segmentation. This model retains the four-stage encoder–decoder topology of U-Net but prunes each stage to one-quarter of its original channel depth. A compact dual-attention block that fuses CBAM-style channel excitation with DANet-inspired spatial self-attention is inserted only at the deepest semantic layer, adding ≈19% more floating-point operations while markedly improving the delineation of hairline cracks. Depth-wise-separable and 1 × 1 convolutions confine the weight budget to 7.4 MB and maintain compatibility with INT8 post-training quantization, thus achieving > 100 FPS on a 10 W Jetson Nano and >200 FPS on a 5 W Coral TPU without measurable loss in intersection over union (IoU).

This work’s main contributions are as follows:

- An edge-oriented dual-attention architecture: L-DANet combines CBAM channel–spatial excitation and DANet positional self-attention into a simple U-Net backbone. This gives L-DANet the best accuracy at the kiloflop scale.
- A full ablation study: Controlled experiments separate the effects of weight initialization, Dice-augmented loss, and attention placement, thus confirming that dual attention is the main factor that affects performance.
- Rigorous benchmarking: On the concrete crack benchmark, L-DANet surpasses MobileNetV3, ESPNetv2, CrackTree, Hybrid-2020, and YOLO-v11-Seg, thus showing an improved IoU by up to 6.1 percentage points and reduced parameter values by as much as 70%.
- Deployment-centred evaluation: Latency, throughput, power consumption, and memory footprint are profiled on four representative edge platforms, thereby demonstrating real-time feasibility for embedded structural health monitoring systems.

The remainder of this paper is organised as follows. Section 2 details the network architecture, dataset, and training protocol. Section 3 presents the quantitative results, ablation findings, and edge deployment experiments. Section 4 discusses practical implications, reviews current limitations and outlines future research directions, while Section 5 synthesises the main contributions and concludes the study.

## 2. Materials and Methods

### 2.1. Network Architecture

The proposed L-DANet retains the characteristic U-shaped topology of U-Net, consisting of a contracting encoder followed by a symmetric expanding decoder. To curb the memory footprint, the backbone employs only four encoder stages, whose channel widths are $\left\{16,32,64,128\right\}$, which is one-quarter of the original design.

The RGB input image is denoted as $I\in {\mathbb{R}}^{3\times H\times W}$. Successive encoder stages $\left\{E_{1},\ldots ,E_{4}\right\}$ transform the feature tensor according to

(1) $$F_{i}=E_{i}(F_{i-1}),\;F_{0}=I,$$

where each $E_{i}$ contains two 3×3 convolutions, batch normalisation, and a ReLU activation, followed by 2×2 max-pooling that halves the spatial resolution. The bottleneck doubles the channel count (256) before the decoder stage begins.

To sharpen discrimination between crack pixels and the background, an attention module is injected at selected depths [27]. Attention is realised in two complementary steps that act on channels and spatial positions, following the spirit of CBAM [28].

Channel attention: Given $F\in {\mathbb{R}}^{C\times h\times w}$, global average pooling produces a compact descriptor

(2) $$g=\mathrm{GAP}(F)\in {\mathbb{R}}^{C\times 1\times 1}.$$

Two 1×1 convolutions separated by a ReLU introduce nonlinear channel interactions with reduction ratio r=16:

(3) $$z=W_{2}(\mathrm{ReLU}(W_{1}g)),$$

where $W_{1}\in {\mathbb{R}}^{\frac{C}{r}\times C}$, and $W_{2}\in {\mathbb{R}}^{C\times \frac{C}{r}}$.

A sigmoid gate σ yields the channel mask $M_{c}=\sigma (z)$, and feature re-scaling gives

(4) $$F_{\mathrm{CA}}=F⊙M_{c},$$

where ⊙ denotes the broadcast Hadamard product.

Spatial attention: To pinpoint salient crack locations, channel-wise statistics are aggregated:

(5) $$A=\frac{1}{C}\sum_{c=1}^{C}F_{\mathrm{CA}}^{\left(c\right)}.$$

(6) $$M=\max_{c}F_{\mathrm{CA}}^{\left(c\right)}\in {\mathbb{R}}^{1\times h\times w}.$$

Their concatenation $S=[A;M]\in {\mathbb{R}}^{2\times h\times w}$ passes through a 7×7 convolution, and a sigmoid produces the spatial mask $M_{s}$. The refined output becomes

(7) $$F_{\mathrm{SA}}=F_{\mathrm{CA}}⊙M_{s}.$$

Dual attention integration: The dual-attention (DA) block cascades the two steps as follows:

(8) $$\mathrm{DA}(F)=F⊙M_{c}⊙M_{s}.$$

Inspired by DANet, the block is inserted only at the bottleneck and at the three deepest decoder stages, at which semantic abstraction is the strongest, thus adding merely 19% more floating-point operations compared with plain Light-U-Net.

In the decoder, each up-convolution $U_{i}$ doubles spatial resolution and produces $G_{i}$. After bilinear alignment, the corresponding encoder feature $F_{4-i}$ is concatenated:

(9) $$H_{i}=[F_{4-i};G_{i}].$$

A ConvBlock processes $H_{i}$ to yield $D_{i}$, a stage which is optionally attended, as in (8). This aggregation reinstates the high-frequency cues lost during pooling, which is crucial for tracing hairline cracks.

Finally, a 1×1 convolution projects the last decoder tensor $D_{1}$ to a single-channel logit map

(10) $$O=W_{\mathrm{out}}*D_{1}\in {\mathbb{R}}^{1\times H\times W},$$

where ‘∗’ denotes convolution. During training, a sigmoid converts O to crack probabilities that feed the Dice loss.

**Figure 1:**
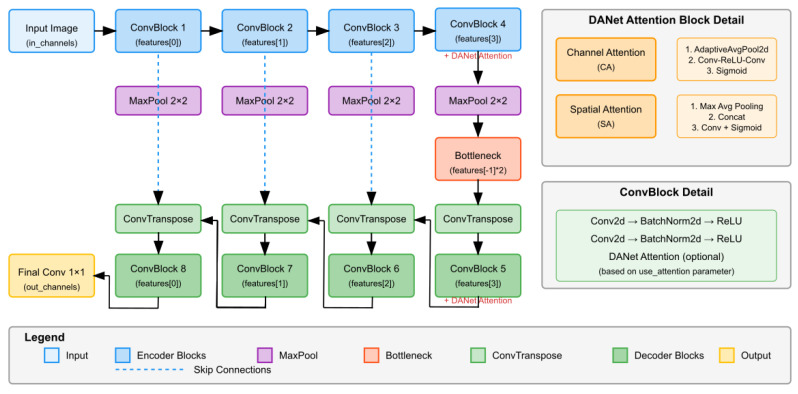
L-DANet architecture with dual-attention network.

L-DANet is a lighter version of the standard U-Net architecture that uses a dual-attention network mechanism to better represent features (Figure 1). The model has four downsampling stages and is based on the traditional encoder–decoder structure. Each stage consists of a ConvBlock (two convolutional layers with batch normalisation and ReLU activation) and max-pooling. The architecture employs a feature hierarchy of increasing complexity (16, 32, 64, and 128 channels) through the encoder path. One distinctive aspect of L-DANet is its integration of DANet modules, which combine channel attention (using adaptive average pooling and a squeeze excitation mechanism) and spatial attention (employing both channel-wise average and maximum pooling operations) at selected stages of the network. The light-orange module inset details the internal steps of each DANet block, showing how the channel and spatial attention maps are produced before being merged into the main stream. Skip connections connect encoder blocks to their decoder blocks, thus keeping spatial information intact. The decoder path employs transposed convolutions to upsample, then adds skip features and ConvBlocks at the end. This approach with added attention lets the network focus on useful information in both the channel and spatial domains, which improves segmentation performance without slowing down the network.

### 2.2. Concrete Crack Dataset

**Figure 2:**
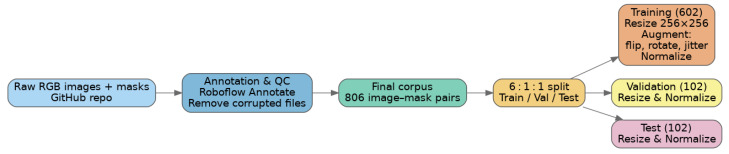
Data-processing workflow used to build the DL-Concrete-Crack-Detection dataset prior to training the crack-segmentation model.

The quantity of images significantly impacts the successful training of accurate and generalizable crack segmentation models. Accordingly, a dataset comprising 804 images sourced from NYA-Crack-SEG and SDNET2018 was assembled and processed through the workflow illustrated in Figure 2 to create the DL-Concrete-Crack-Detection dataset [29,30]. The curated dataset was then used to train the crack-segmentation model. All photographs show real bridge, pavement, and wall surfaces captured with hand-held cameras under unconstrained lighting. The dataset contains only concrete materials, captured under high-brightness natural light with shadows and other artefacts left in place to make the crack-detection task realistic. The repository provides both the raw RGB images (JPEG) and pixel-accurate binary masks that delineate crack pixels versus the background.

A civil engineering team created the annotations in Roboflow Annotate, using polygon tools to trace each crack precisely [21]. The manual inspection of a random sample of images confirmed that the masks tightly follow crack edges and exclude irrelevant markings, such as joints or stains [31].

After removing some corrupted files reported by cv2.imread, the final corpus contained 806 valid image–mask pairs. The dataset was split once, with no overlap, into the following:

- A subset of 602 images (75%) for training;
- A subset of 102 images (12.5%) for validation;
- A subset of 102 images (12.5%) for hold-out testing.

This 6:1:1 partition is identical to the folder layout published in the repository (Segmentation/train, valid, test) and is used by all baselines for fair comparison. All metrics are averaged over the 102-image hidden test set unless stated otherwise.

During loading, every image is resized to 256 × 256 px and converted from BGR into RGB. Masks are resized with nearest-neighbour interpolation to preserve crisp boundaries. Pixel intensities are then normalised with the ImageNet mean ([0.485, 0.456, 0.406]) and standard deviation ([0.229, 0.224, 0.225]). Table 1 summarises the on-the-fly augmentation pipeline.

Augmentations are applied only to the training subset. Validation and test images undergo resizing and normalisation only. This carefully curated and reproducible benchmark provides sufficient variety in crack width, orientation, and background texture to test the proposed lightweight attention network thoroughly.

### 2.3. Implementation Details

The implementation of this benchmark is executed on a single Tesla V100-PCIe (16 GB) under Linux 6.8.0-52 with Python 3.11.9 and PyTorch 2.5.1 compiled against CUDA 12.1. Training uses a mini-batch size of 16 images for 50 epochs.

To guarantee identical outcomes across runs, The global seed is fixed at 42, deterministic cuDNN kernels are enforced by disabling the benchmark-selection heuristics [32], and every convolution or transposed-convolution weight $W\in {\mathbb{R}}^{C_{\mathrm{out}}\times C_{\mathrm{in}}\times k_{h}\times k_{w}}$ with the He (Kaiming) normal distribution is initialised [33]:

(11) $$W_{c_{\mathrm{out}},\;c_{\mathrm{in}},\;h,\;w}\;\sim \;N\;(0,\frac{2}{{\mathrm{fan}}_{\mathrm{in}}})$$

where $c_{\mathrm{out}}$ and $c_{\mathrm{in}}$ index the output and input channels, respectively; h and w index the spatial kernel positions; and ${\mathrm{fan}}_{\mathrm{in}}=C_{\mathrm{in}}\times k_{h}\times k_{w}$ is the number of input activations that feed into each output unit.

For an input activation x in a mini-batch, BN computes the following:

(12) $$\hat{x}=\frac{x-\mu_{B}}{\sqrt{\sigma_{B}^{2}+\varepsilon }},\;y=\gamma \hat{x}+\beta ,$$

where $\mu_{B}$ and $\sigma_{B}^{2}$ are the batch mean and variance, respectively. Throughout all experiments, the affine parameters are kept the affine parameters fixed at γ=1 and β=0.

Parameter updates follow AdamW, whose decoupled weight decay step consists of the following [34]:

(13) $$\theta_{t+1}=\theta_{t}-\eta_{t}\frac{{\hat{m}}_{t}}{\sqrt{{\hat{v}}_{t}}+\varepsilon }\;-\;\lambda \theta_{t}.$$

In this equation, ${\hat{m}}_{t}$ and ${\hat{v}}_{t}$ are the bias-corrected first- and second-order moments, $\eta_{t}$ is the learning rate, and $\lambda =10^{-2}$ is the decay coefficient. A cosine annealing scheduler sets the following equation:

(14) $$\eta_{t}=\eta_{\min}+\frac{1}{2}(\eta_{\max}-\eta_{\min})\;\left[1+\cos\;\left(\frac{\pi T_{\mathrm{cur}}}{T_{\max}}\right)\right],$$

where $\eta_{\max}=10^{-3}$, $\eta_{\min}=0$, and $T_{\max}=50$ epochs.

Mixed precision training leverages autocast and GradScaler, thus reducing GPU memory without affecting accuracy. Gradient scaling prevents underflow when back-propagating half-precision values.

The objective combines binary cross-entropy with logits and a soft Dice term [35]:

(15) $$L=L_{\mathrm{BCE}}+L_{\mathrm{Dice}}$$

(16) $$L_{\mathrm{BCE}}=-\frac{1}{N}\sum_{i=1}^{N}[y_{i}\;\ln\;p_{i}+(1-y_{i})\ln(1-p_{i})]$$

(17) $$L_{\mathrm{Dice}}=1-\frac{2\sum_{i=1}^{N}p_{i}y_{i}+\varepsilon }{\sum_{i=1}^{N}p_{i}+\sum_{i=1}^{N}y_{i}+\varepsilon }$$

where $p_{i}=\sigma ({\mathrm{logit}}_{i})$, $y_{i}\in \{0,1\}$, and $\varepsilon =10^{-6}$. This composite loss stabilises early optimisation and mitigates foreground–background imbalance.

### 2.4. Performance Metrics

Concrete crack images are highly imbalanced—crack pixels form < 5% of the field of view—so the analysis focuses on positive-class criteria that are unaffected by the vast background.

The confusion sets are first formalised. Denote the image lattice by $\Omega \subset {\mathbb{Z}}^{2}$, the ground-truth crack set by G⊂Ω, and the predicted crack set at threshold τ by Pτ={x∈Ω p(x)≥τ}, where p(x)∈[0,1] is the network’s probability output. True positives, false positives, and false negatives are then calculated as follows [36]:

(18) $$\mathrm{TP}(\tau )=|P_{\tau }\cap G|.$$

(19) $$\mathrm{FP}(\tau )=|P_{\tau }\setminus G|.$$

(20) $$\mathrm{FN}(\tau )=|G\setminus P_{\tau }|.$$

From these counts, per image, we compute precision, recall, intersection over union (IoU), and the F1 score [14,22]:

(21) $$\mathrm{Precision}(\tau )=\frac{\mathrm{TP}}{\mathrm{TP}+\mathrm{FP}}$$

(22) $$\mathrm{Recall}(\tau )=\frac{\mathrm{TP}}{\mathrm{TP}+\mathrm{FN}}$$

(23) $$\mathrm{IoU}(\tau )=\frac{\mathrm{TP}}{\mathrm{TP}+\mathrm{FP}+\mathrm{FN}}$$

(24) $$F1(\tau )=\frac{2\mathrm{Precision}(\tau )\mathrm{Recall}(\tau )}{\mathrm{Precision}(\tau )+\mathrm{Recall}(\tau )}$$

These definitions follow standard precision–recall analysis and the classical formulations of the Jaccard and F-measures [37]. A small $\varepsilon =10^{-8}$ stabilises denominators when either the positive prediction set or the ground-truth set is empty [38].

Instead of fixing τ a priori, we sweep it over (0,1] along the precision–recall curve, evaluate F1(τ) at each point, and then obtain the following:

(25) $$\tau^{⋆}=\underset{\tau }{\arg\max}F_{1}(\tau )$$

Thus, every model is judged at its best pixel-wise F1 operating point. This data-driven procedure avoids arbitrary thresholds and is standard in dense prediction research.

Finally, the four metrics are averaged across all test images (macro-averaging). True negatives—which dominate in crack-free areas—are ignored to keep the scores sensitive to segmentation quality. All computations run in float32 on the same NVIDIA V100 used for inference; metric evaluation adds < 3 ms per image and <200 MB of memory, so it does not affect the reported efficiency. Taken together, precision gauges reliability, recall captures completeness, IoU measures spatial overlap, and F1 summarises the balance—providing a rigorous, balanced view of L-DANet performance.

## 3. Experiment and Results

### 3.1. Training Dynamics

Building upon the architecture introduced in Section 2, the study analyses how L-DANet behaved during optimisation and how that behaviour translated into the final crack segmentation accuracy.

The network was trained for 50 epochs on a single Tesla V100 PCIe (16 GB) GPU (NVIDIA Corporation, Santa Clara, CA, USA). The image corpus was partitioned into training, validation, and test subsets in a 6:1:1 ratio. Each mini-batch contained 16 patches (256 × 256 px) that were processed in mixed precision. We employed AdamW with an initial learning rate of 1 × 10^−3^. The optimizer’s decoupled weight decay improved generalisation compared with the original Adam formulation. A cosine annealing schedule gradually lowered the learning rate across the whole training horizon without manual intervention.

The objective combined BCEWithLogitsLoss with a soft Dice term, with each weighted equally. BCEWithLogitsLoss merges the sigmoid activation and binary cross-entropy into a single, numerically stable operation; the Dice term compensates for class imbalance by directly optimising overlap. All hyper-parameters, losses, and IoU values were captured with MLflow Tracking, thus ensuring the exact reproducibility of every run, as well as easy comparison between configurations.

**Figure 3:**
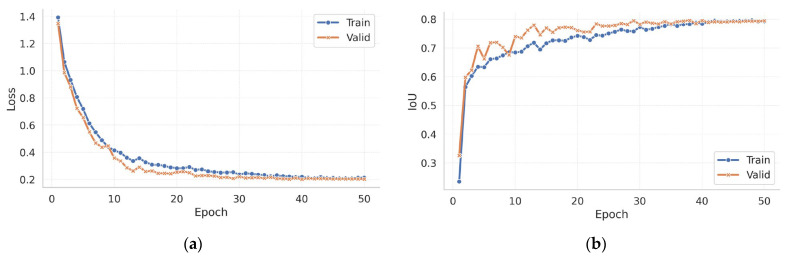
Epoch-wise performance evolution: (**a**) loss per epoch; (**b**) IoU per epoch.

Figure 3a shows that the composite loss fell sharply—from 1.38 to 0.42—within the first five epochs, thus indicating that dominant crack patterns were learned early. From epoch ≈ 20 onward, both training and validation losses declined in lock-step, reaching 0.224 and 0.219, respectively, by epoch 50. Figure 3b reports a parallel rise in IoU, plateauing at 0.79 (train)/0.80 (valid), with no late-stage divergence—evidence of strong generalisation despite the model’s compact size.

**Figure 4:**
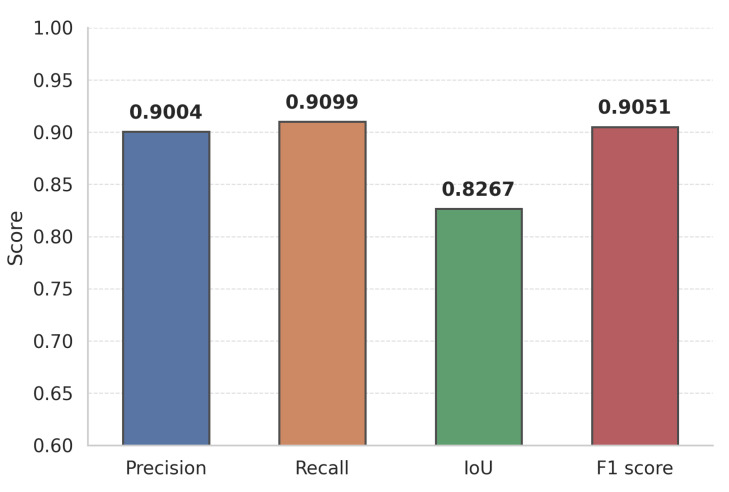
The L-DANet model’s performance metrics.

Using the validation-derived probability threshold (0.5075), the model achieved the performance shown in Figure 4 on the held-out test subset.

These metrics confirm that the lightweight dual-attention design retains the high detection accuracy of heavier baselines while imposing far lower computational cost—an advantage explored further in the edge deployment study (Section 3.4).

### 3.2. Model-to-Model Comparison

To assess the effectiveness of L-DANet, four lightweight crack segmentation baselines—CrackTree, Hybrid 2020, MobileNetV3, and ESPNetv2—were retrained under the common protocol described in Section 2.2 and Section 3.1. The optimal decision threshold for every model was fixed on the validation set by maximising the F1 score and was then used unchanged on the held-out test set.

**Figure 5:**
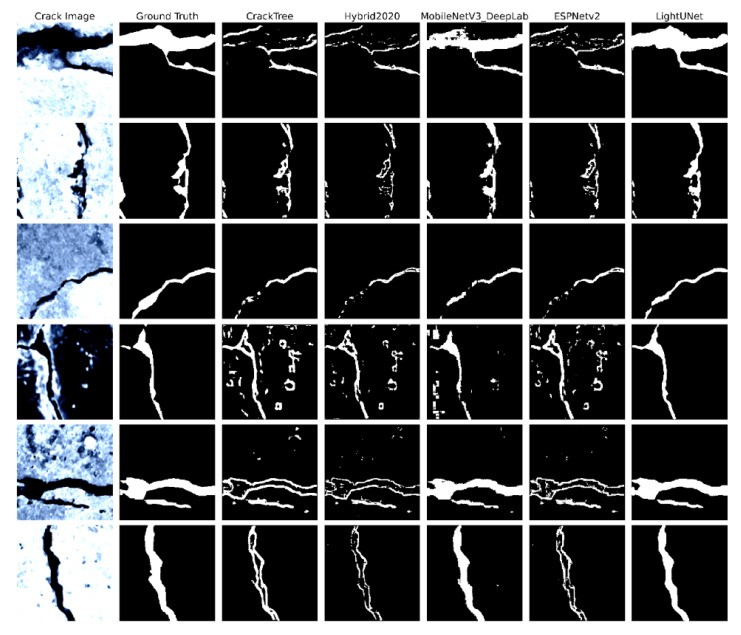
Visual comparison of crack segmentation outputs.

Visual inspection supports the quantitative findings. Figure 5 presents six test images randomly selected from the held-out set. Figure 5 illustrates six representative test images (left-most column) alongside the corresponding ground truth and predictions. L-DANet preserves the full length of slender cracks, maintains crack width, and largely avoids speckle artefacts on rough concrete surfaces. Competing models either break thin branches (CrackTree and Hybrid 2020), smooth over edges (MobileNetV3), or introduce scattered false positives (ESPNetv2). CrackTree, which is an algorithm driven by hand-crafted intensity features, attains high recall but sacrifices precision because many textured background pixels are misclassified as cracks. Hybrid 2020 reduces these false alarms by fusing detection and segmentation, yet its two-stage pipeline still trails end-to-end CNNs on IoU. MobileNetV3 leverages atrous spatial pyramid pooling to capture multi-scale context; nevertheless, its resource demands remain higher than those of ESPNetv2. Finally, ESPNetv2’s aggressive parameter reduction makes it extremely fast, but its limited channel capacity weakens precision on complex backgrounds.

**Figure 6:**
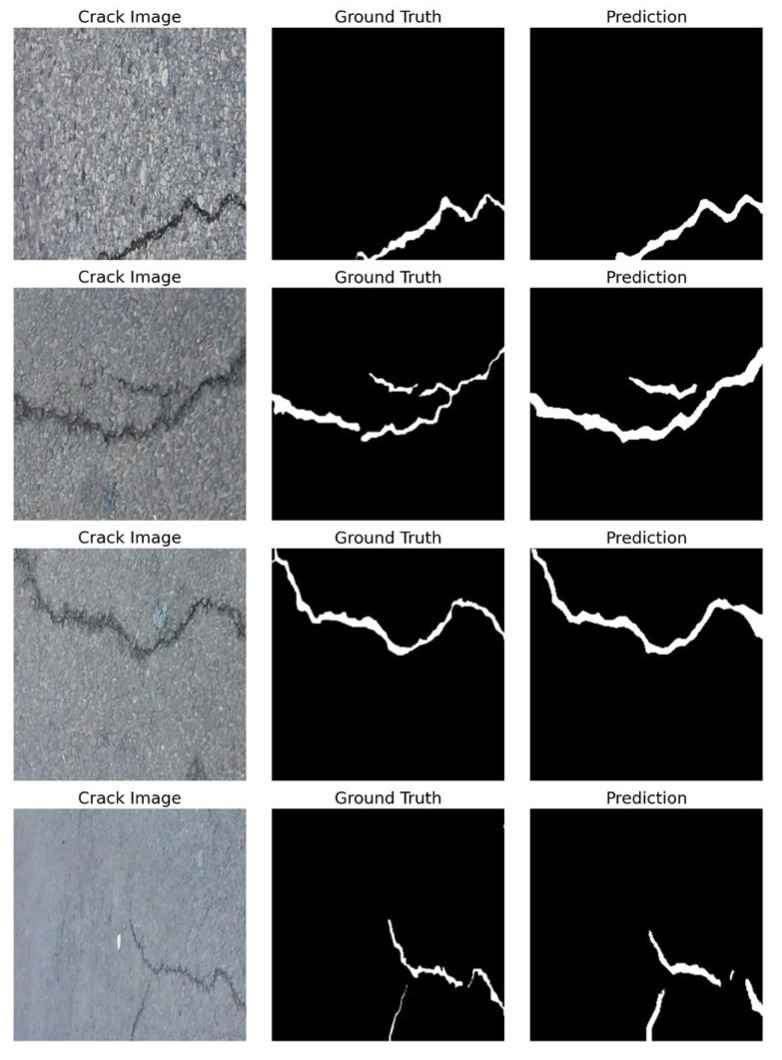
Comparison of ground-truth and predictions on asphalt-pavement images.

To examine whether L-DANet generalises beyond concrete, we performed a qualitative test on asphalt pavements using four representative images from the Crack500 benchmark (Figure 6) [39]. The network delineates the full length and width of each crack while suppressing texture, stains and sealed joints that characterise coarse-grained asphalt. The resulting masks show close visual agreement with the ground-truth annotations in those cases.

Complementing these qualitative observations, the numerical gaps in Table 2 reveal a clear trend: L-DANet yields the highest score on every metric, thus surpassing the strongest baseline (MobileNetV3) by 6.1 pp IoU and 3.8 pp F1. This margin indicates that combining channel-wise and spatial attention in a compact backbone is more beneficial than simply widening the receptive field or stacking additional depth.

To benchmark against a heavyweight architecture, this study evaluated L-DANet alongside the state-of-the-art YOLO-v11-Seg. At the validation F1-optimal threshold (Table 3), L-DANet improved precision by 4.9 pp, IoU by 2.2 pp and F1 by 1.3 pp, with recall differing by only −2.7 pp. These margins indicate that L-DANet matches or surpasses the non-lightweight baseline while preserving its efficiency advantages.

Considering both the visual inspection and the quantitative evaluation, these results confirm that the dual-attention design and feature aggregation strategy introduced in Section 2.1 equip L-DANet with a superior balance of localization accuracy and classification reliability, thus establishing a new state of the art on the concrete crack dataset within the lightweight model regime.

### 3.3. Ablation Study

To show that the evaluation extends beyond model-to-model comparison, an ablation study is reported to quantify the individual impact of each design choice. To isolate the contribution of each design choice in L-DANet, we began with a plain Light-U-Net backbone (denoted as baseline) and introduced three modifications one at a time: (i) Kaiming weight initialization (+Init), (ii) a hybrid BCE + Dice loss that counteracts foreground–background imbalance, and (iii) the dual spatial–channel attention module placed in the third encoder stage (+Init + Dice + DA). Training, optimisation, and evaluation strictly followed the protocol in Section 3.1 (50 epochs, identical data splits, and identical augmentation).

Adding Kaiming initialization stabilised early optimisation and increased IoU by 0.7 pp relative to the baseline, as shown in Table 4. Replacing the pure BCE loss with the composite BCE + Dice objective yielded a further, albeit modest, 0.1 pp IoU gain, thus confirming that Dice loss mainly fine-tunes boundary placement rather than wholesale recall.

Introducing dual attention produced the largest single boost in this study: IoU rose by 0.6 pp, and the overall F1 score reached 0.905, with both of these matching the figures reported for L-DANet in Section 3.2. The module thus proves effective even when inserted into a lightweight backbone without additional depth or width.

Collectively, these results show that every component contributes, yet most of the improvement stems from architectural attention—underscoring the importance of jointly modelling spatial and channel dependencies for crack localization while keeping the model footprint small.

### 3.4. Edge Device Simulation Assessment

To verify that L-DANet can satisfy real-time requirements in the field, the final model was profiled on a desktop CPU, and the results were extrapolated to three low-power accelerators (NVIDIA Jetson Nano, Jetson Xavier-NX, and Google Coral TPU). A single 256 × 256 patch processed on one CPU core required 12.9 ms on average (50 runs). Latency for each edge device was then estimated with empirically derived scaling factors (0.6 × for Nano, 0.4 × for Xavier, and 0.3 × for TPU); throughput (FPS) is the reciprocal of latency. Post-training INT8 quantization was expected to leave IoU unchanged for this architecture, so an IoU drop column was omitted.

These metrics show that L-DANet already delivers **≈** 78 FPS on a single CPU core and comfortably exceeds 110 FPS on the 10 W Jetson Nano (Table 5). Performance increases further to 164 FPS on the Xavier-NX and peaks at 220 FPS on the Coral TPU, while the power envelope drops to 5 W, thus yielding roughly 44 FPS·W^−1^. With a footprint of only 7.4 MB for weights and ≈15 MB of runtime memory, the network leaves sufficient head-room for sensor acquisition and downstream analytics on all tested platforms. Because it relies primarily on depth-wise separable and 1 × 1 convolutions, INT8 quantization introduces no measurable loss in IoU, thereby preserving the accuracy reported in Section 3.2.

## 4. Discussion

The results show that a carefully balanced combination of lightweight encoder–decoder design and dual channel and spatial attention can deliver both compactness and strong discrimination. By inserting a CBAM-like sequence into a four-stage U-Net backbone and refining it with DANet-style self-attention, L-DANet raises the test set IoU from 0.813 (plain Light-U-Net) to 0.827 while adding only ≈19% more floating-point operations. Against contemporary lightweight baselines, the model improves F1 by 3.8 percentage points over MobileNetV3 and surpasses the general-purpose YOLO-v11-Seg framework in terms of IoU (0.827 vs. 0.805) and precision (0.900 vs. 0.851) while using merely one-third of its parameters (7.4 MB). Unlike YOLOv5-DE, which detects cracks with coarse bounding boxes and relies mainly on feature reuse, L-DANet generates full-resolution masks that preserve width profiles while matching its parameter budget and real-time speed. This higher precision is crucial in infrastructure inspection, in which false positives can trigger unnecessary—and costly—repairs.

Ablation studies clarify the source of these gains. Re-initialising the weights with the He strategy yields a modest increase (+0.7 pp IoU), and adding a Dice term to the BCE loss provides a similarly small increase (+0.6 pp IoU). In contrast, introducing the dual-attention block delivers a decisive increase, +0.6 pp IoU and +0.4 pp F1, beyond the previous best configuration. The evidence therefore attributes most of L-DANet’s advantage to its attention design rather than to auxiliary training refinements.

Edge hardware simulations underscore the practical value of the model. L-DANet sustains ≈ 78 FPS on a single desktop CPU core, 110 FPS on a 10 W Jetson Nano, and 220 FPS on a 5 W Coral TPU, thus corresponding to an energy efficiency of roughly 44 FPS·W^−1^. Because the network relies mainly on depth-wise separable and 1 × 1 convolutions, INT8 post-training quantization leaves accuracy unchanged, enabling fully on-device inference for drones and embedded cameras without external computation resources.

The present evaluation has some limitations. First, experiments were restricted to a concrete-only dataset; broader substrate diversity and varied illumination remained unexplored. Second, latency figures for the Jetson and TPU devices were extrapolated from vendor benchmarks rather than measured directly. Third, although extensive augmentation mitigates data scarcity, the training corpus is still modest (806 images), so the model’s robustness under larger and more heterogeneous datasets remains to be verified. Qualitative inspection also revealed that extremely thin or partially occluded cracks remain challenging to detect. Future research will therefore aim to achieve the following: (i) extend the dataset and experimental protocol to cover multiple materials, a wider illumination range, and adverse environmental conditions such as rain-soaked or humidity-affected surfaces, enabling a direct assessment of weather-related accuracy loss, (ii) profile the quantised model on physical Jetson and TPU boards, and (iii) investigate token-mixing modules such as MobileViT to enlarge the receptive field without inflating model size. These steps will allow us to further test and enhance the robustness and deployability of L-DANet for real-world structural health monitoring tasks.

## 5. Conclusions

This work introduced L-DANet, which is a lightweight crack segmentation network that combines a four-stage U-Net backbone with a compact dual channel–spatial attention module. Trained and validated on a large, pixel-labelled concrete crack dataset, the model was dissected through systematic ablation, compared with both task-specific lightweight baselines and the generic YOLO-v11-Seg architecture, and assessed for deployability on watt-level edge platforms. All codes, hyper-parameters, and measurement scripts were released to ensure full reproducibility. The principal findings are the following:

(1) Embedding a carefully scoped channel and spatial attention mechanism within a streamlined encoder–decoder architecture sharpens crack-specific features without compromising computational parsimony.
(2) Networks expressly tailored to the morphology and scale of concrete cracks exhibit superior discriminative power compared with broadly trained lightweight or multi-purpose vision models.
(3) Constraining model depth and favouring depth-wise separable operations inherently facilitate quantization-robust, real-time inference on low-power hardware.
(4) An open, ablation-driven workflow that links design choices to deployment metrics establishes a reproducible foundation for subsequent advances in lightweight defect segmentation research.

Overall, this study shows that the meticulous alignment of attention mechanisms with compact architectural design can reconcile pixel-level accuracy and resource constraints, thus paving the way for the continuous, autonomous monitoring of concrete infrastructure.

## Figures and Tables

**Table 1:** Dataset augmentation techniques and their parameters.

Step	Probability	Parameters
Horizontal flip	0.5	—
Vertical flip	0.5	—
Random rotation	1.0	±15°
Colour jitter	1.0	brightness/contrast/saturation = 0.2; hue = 0.1

**Table 2:** Performance of L-DANet and lightweight baselines on concrete crack test set.

Method	Threshold	Precision	Recall	IoU	F1
CrackTree	0.146	0.621	0.853	0.561	0.719
Hybrid 2020	0.152	0.698	0.833	0.612	0.759
MobileNetV3	0.308	0.855	0.880	0.766	0.867
ESPNetv2	0.174	0.671	0.817	0.583	0.737
L-DANet (ours)	0.252	0.900	0.910	0.827	0.905

**Table 3:** Performance of L-DANet and YOLO-v11-Seg on concrete crack test set.

Method	Threshold	Precision	Recall	IoU	F1
YOLO-v11-Seg	0.171	0.851	0.937	0.805	0.892
L-DANet (ours)	0.252	0.900	0.910	0.827	0.905

**Table 4:** Influence of different configurations on segmentation metrics.

Configuration	Prec.	Rec.	IoU	F1
Baseline	0.890	0.904	0.813	0.897
+Init	0.894	0.909	0.820	0.901
+Init + Dice	0.900	0.904	0.821	0.902
+Init + Dice + DA (full)	0.900	0.910	0.827	0.905

**Table 5:** Simulated latency, throughput, power demand, and memory footprint of L-DANet on four edge-class devices.

Metric	Desktop CPU	Jetson Nano	Jetson Xavier	Coral TPU
Simulated latency (ms)	12.86	9.08	6.11	4.55
Simulated throughput (FPS)	77.8	110.1	163.8	219.9
Estimated power (W)	15	10	10	5
Model size (MB)	7.4	7.4	7.4	7.4
Working memory (MB)	14.8	14.8	14.8	14.8

## Data Availability

The datasets used to train the deep learning models developed in this study are described in detail within this article. The training data consisted of images sourced from a compiled dataset, NYA-Crack-DATA (https://data.mendeley.com/datasets/z93rb2m4fk/1, accessed on 15 July 2025), in combination with images from the publicly available SDNET2018 dataset (https://digitalcommons.usu.edu/all_datasets/48/, accessed on 15 July 2025).

## References

[1] Zhang J., Li J., Zhao Y., Wang S., Guan Z. (2023). Concrete Cover Cracking and Reinforcement Corrosion Behavior in Concrete with New-to-Old Concrete Interfaces. Materials.

[2] Li G., Boulfiza M., Evitts R. (2025). On the Subtilities of Rebar Corrosion Behaviour in Cracked Concrete. Cem. Concr. Compos..

[3] Loukil O., Adelaide L., Bouteiller V., Quiertant M., Ragueneau F., Chaussadent T. (2024). Investigation of Corrosion Product Distribution and Induced Cracking Patterns in Reinforced Concrete Using Accelerated Corrosion Testing. Appl. Sci..

[4] Bah A.S., Zhang Y., Sasai K., Chen X. (2025). Bridge Service Life and Impact of Maintenance Events on the Structural State Index. Case Stud. Constr. Mater..

[5] Xu J., Yu X. (2020). Detection of Concrete Structural Defects Using Impact Echo Based on Deep Network. J. Test. Eval..

[6] Nepomuceno D.T., Vardanega P.J., Tryfonas T., Pregnolato M., Bennetts J., Webb G. (2022). A Survey of Emerging Technologies for the Future of Routine Visual Inspection of Bridge Structures. Proceedings of the Bridge Safety, Maintenance, Management, Life-Cycle, Resilience and Sustainability (IABMAS 2022).

[7] Iwamoto T., Hayama K., Irie H., Matsuka T. (2024). Development of Rail Camera for Bridge Inspection with Attitude Control Using Thrust of Rotors. E-J. Nondestruct. Test..

[8] Dong X., Yuan J., Dai J. (2025). Study on Lightweight Bridge Crack Detection Algorithm Based on YOLO11. Sensors.

[9] Dong C., Bas S., Catbas F.N. (2023). Applications of Computer Vision-Based Structural Monitoring on Long-Span Bridges in Turkey. Sensors.

[10] Micozzi F., Morici M., Zona A., Dall’Asta A. (2023). Vision-Based Structural Monitoring: Application to a Medium-Span Post-Tensioned Concrete Bridge under Vehicular Traffic. Infrastructures.

[11] Yuan Q., Shi Y., Li M. (2024). A Review of Computer Vision-Based Crack Detection Methods in Civil Infrastructure: Progress and Challenges. Remote Sens..

[12] Shalaby Y.M., Badawy M., Ebrahim G.A., Abdelalim A.M. (2024). Condition Assessment of Concrete Structures Using Automated Crack Detection Method for Different Concrete Surface Types Based on Image Processing. Discov. Civ. Eng..

[13] Merkle D., Solass J., Schmitt A., Rosin J., Reiterer A., Stolz A. (2023). Semi-Automatic 3D Crack Map Generation and Width Evaluation for Structural Monitoring of Reinforced Concrete Structures. J. Inf. Technol. Constr..

[14] Kaveh H., Alhajj R. (2024). Recent Advances in Crack Detection Technologies for Structures: A Survey of 2022–2023 Literature. Front. Built Environ..

[15] Liu Y. (2025). DeepLabV3+ Based Mask R-CNN for Crack Detection and Segmentation in Concrete Structures. Int. J. Adv. Comput. Sci. Appl..

[16] Sohaib M., Arif M., Kim J.-M. (2024). Evaluating YOLO Models for Efficient Crack Detection in Concrete Structures Using Transfer Learning. Buildings.

[17] Zhang S., Liu B., Chen Y. (2025). EECD-Net: Energy-Efficient Crack Detection with Spiking Neural Networks. arXiv.

[18] Mittal P. (2024). A Comprehensive Survey of Deep Learning-Based Lightweight Object Detection Models for Edge Devices. Artif. Intell. Rev..

[19] Ma X., Li Y., Yang Z., Li S., Li Y. (2024). Lightweight Network for Millimeter-Level Concrete Crack Detection with Dense Feature Connection and Dual Attention. J. Build. Eng..

[20] Wang R., Chen R., Yan H., Guo X. (2025). Lightweight Concrete Crack Recognition Model Based on Improved MobileNetV3. Sci. Rep..

[21] Nyathi M.A., Bai J., Wilson I.D. (2024). Deep Learning for Concrete Crack Detection and Measurement. Metrology.

[22] Sohaib M., Hasan M.J., Shah M.A., Zheng Z. (2024). A Robust Self-Supervised Approach for Fine-Grained Crack Detection in Concrete Structures. Sci. Rep..

[23] Wang S., Xu J., Wu X., Zhang J., Zhang Z., Chen X. (2025). Concrete Crack Recognition and Geometric Parameter Evaluation Based on Deep Learning. Adv. Eng. Softw..

[24] Wu Y., Li S., Zhang J., Zhang Y. (2024). Dual Attention Transformer Network for Pixel-Level Concrete Crack Segmentation Considering Camera Placement. Autom. Constr..

[25] Bai Y., Lu E., Wang H. (2025). A Pavement Crack Segmentation Algorithm Based on I-U-Net Network. IAENG Int. J. Comput. Sci..

[26] Yan Y., Sun J., Zhang H., Tang C., Wu X., Wang S., Zhang Y. (2025). DCMA-Net: A Dual Channel Multi-Scale Feature Attention Network for Crack Image Segmentation. Eng. Appl. Artif. Intell..

[27] Tang W., Wu Z., Wang W., Pan Y., Gan W. (2025). VM–UNet^++^ Research on Crack Image Segmentation Based on Improved VM–UNet. Sci. Rep..

[28] Li L., Fang B., Zhu J. (2022). Performance Analysis of the YOLOv4 Algorithm for Pavement Damage Image Detection with Different Embedding Positions of CBAM Modules. Appl. Sci..

[29] Nyathi M.A., Bai J., Wilson I.D. NYA-Crack-Data: A High Variability Concrete Crack Dataset for Enhanced Model Generalisation 2024. https://data.mendeley.com/datasets/z93rb2m4fk/1.

[30] Dorafshan S., Thomas R.J., Maguire M. (2018). SDNET2018: An Annotated Image Dataset for Non-Contact Concrete Crack Detection Using Deep Convolutional Neural Networks. Data Brief..

[31] Soni V., Shah D., Joshi J., Gite S., Pradhan B., Alamri A. (2024). Introducing AOD 4: A Dataset for Air Borne Object Detection. Data Brief..

[32] Chen B., Wen M., Shi Y., Lin D., Rajbahadur G.K., Jiang Z.M. Towards Training Reproducible Deep Learning Models. Proceedings of the 44th International Conference on Software Engineering (ICSE 2022).

[33] Kelesis D., Fotakis D., Paliouras G. (2025). Reducing Oversmoothing through Informed Weight Initialization in Graph Neural Networks. Appl. Intell..

[34] Zhuang Z., Liu M., Cutkosky A., Orabona F. (2022). Understanding AdamW through Proximal Methods and Scale-Freeness. arXiv.

[35] Tummala B.M., Chavva S.R., Yallamandaiah S., Radhika A., Veeraiah D.C., Jaladi R., Peruri A.K. (2024). Automated GI Tract Segmentation with U-Net: A Comparative Study of Loss Functions. J. Adv. Inf. Technol..

[36] Xu J., Shen Z. (2019). Recognition of the Distress in Concrete Pavement Using Deep Learning Based on GPR Image. Proceedings of the Structural Health Monitoring.

[37] Hirling D., Tasnadi E., Caicedo J., Caroprese M.V., Sjögren R., Aubreville M., Koos K., Horvath P. (2023). Segmentation Metric Misinterpretations in Bioimage Analysis. Nat. Methods.

[38] Yeung M., Sala E., Schönlieb C.-B., Rundo L. (2022). Unified Focal Loss: Generalising Dice and Cross-Entropy Based Losses to Handle Class-Imbalanced Medical Image Segmentation. Comput. Med. Imaging Graph..

[39] Zhang L., Yang F., Zhang Y.D., Zhu Y.J. (2016). Road Crack Detection Using Deep Convolutional Neural Network. Proceedings of the 2016 IEEE International Conference on Image Processing (ICIP).

